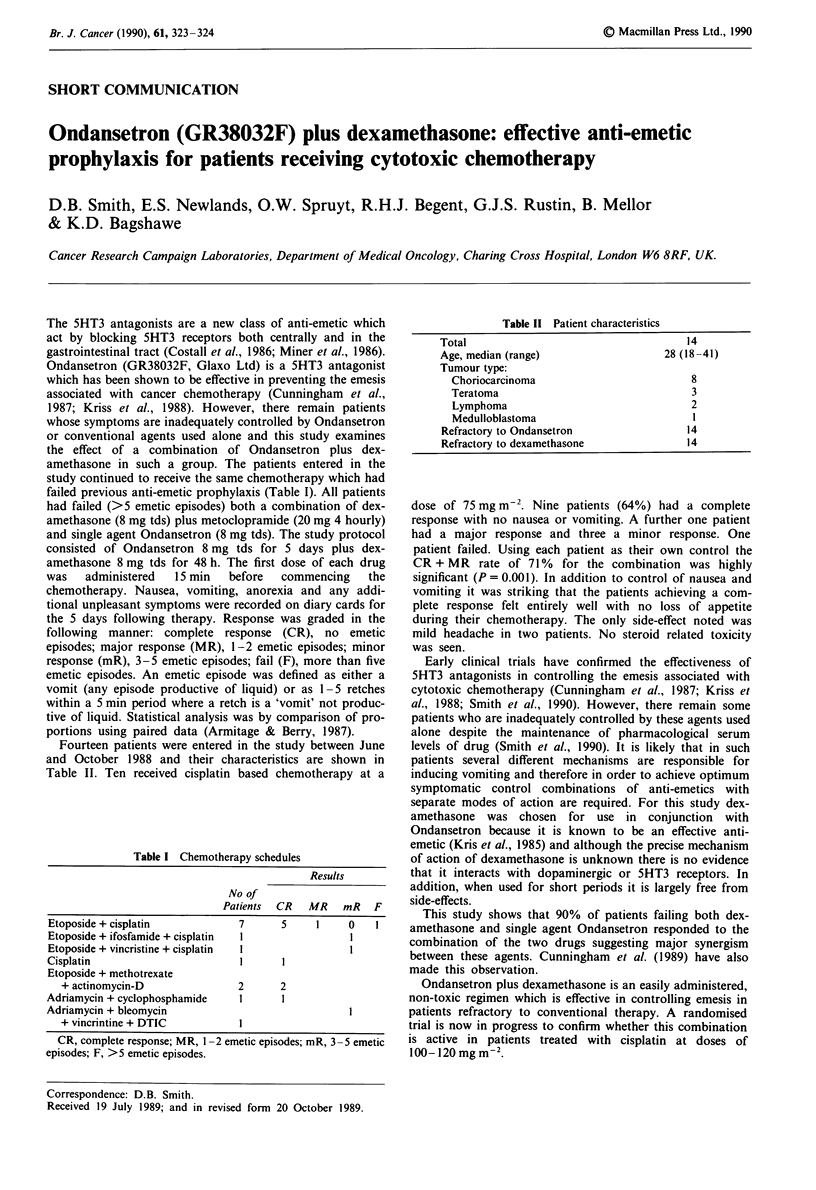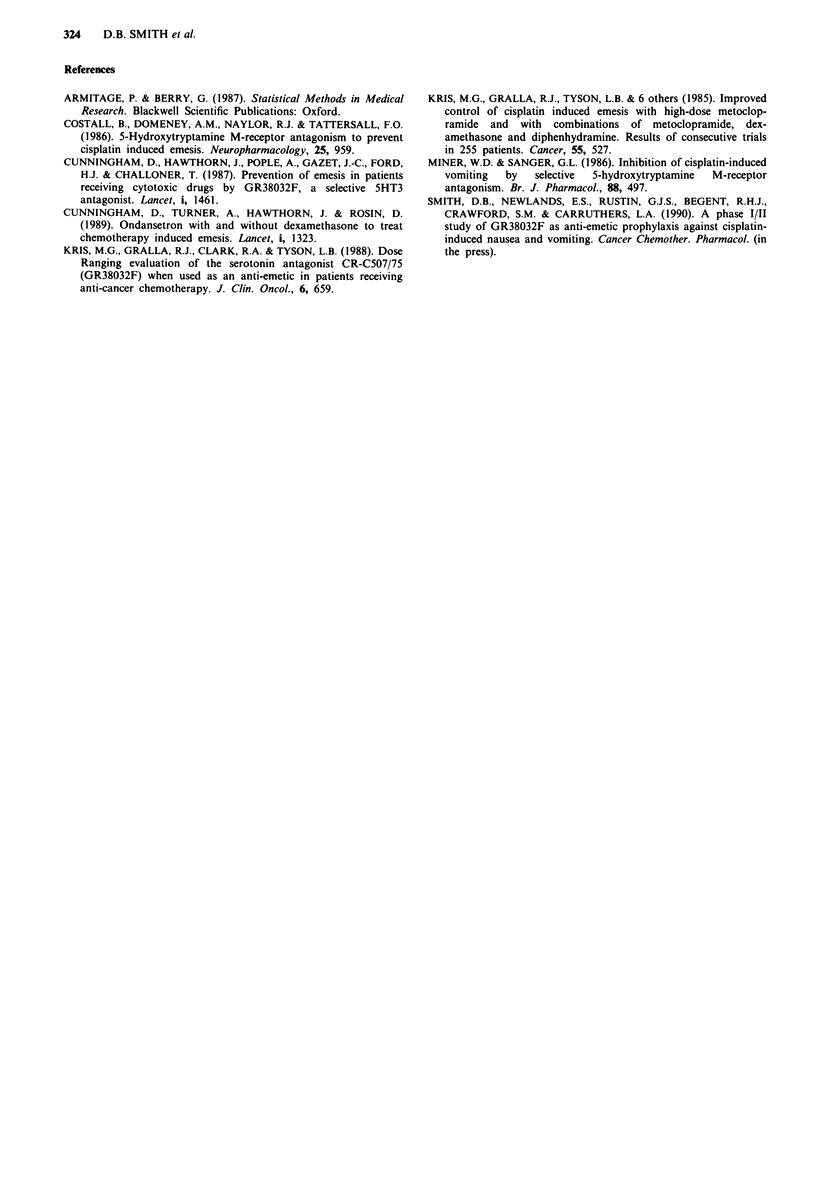# Ondansetron (GR38032F) plus dexamethasone: effective anti-emetic prophylaxis for patients receiving cytotoxic chemotherapy.

**DOI:** 10.1038/bjc.1990.62

**Published:** 1990-02

**Authors:** D. B. Smith, E. S. Newlands, O. W. Spruyt, R. H. Begent, G. J. Rustin, B. Mellor, K. D. Bagshawe

**Affiliations:** Cancer Research Campaign Laboratories, Department of Medical Oncology, Charing Cross Hospital, London, UK.


Br. J. Cancer (1990), 61, 323-324 © Macmillan Press Ltd., 1990

SHORT COMMUNICATION

### Ondansetron (GR38032F) plus dexamethasone: effective anti-emetic prophylaxis for patients receiving cytotoxic chemotherapy

D.B. Smith, E.S. Newlands, O.W. Spruyt, R.H.J. Begent, G.J.S. Rustin, B. Mellor & K.D. Bagshawe

*Cancer Research Campaign Laboratories, Department of Medical Oncology, Charing Cross Hospital, London W6 8RF, UK.*

The 5HT3 antagonists are a new class of anti-emetic which act by blocking 5HT3 receptors both centrally and in the gastrointestinal tract (Costall *et al.*, 1986; Miner *et al.*, 1986). Ondansetron (GR38032F, Glaxo Ltd) is a 5HT3 antagonist which has been shown to be effective in preventing the emesis associated with cancer chemotherapy (Cunningham *et al.*, 1987; Kriss *et al.*, 1988). However, there remain patients whose symptoms are inadequately controlled by Ondansetron or conventional agents used alone and this study examines the effect of a combination of Ondansetron plus dexamethasone in such a group. The patients entered in the study continued to receive the same chemotherapy which had failed previous anti-emetic prophylaxis (Table I). All patients had failed (>5 emetic episodes) both a combination of dexamethasone (8 mg tds) plus metoclopramide (20 mg 4 hourly) and single agent Ondansetron (8 mg tds). The study protocol consisted of Ondansetron 8 mg tds for 5 days plus dexamethasone 8 mg tds for 48 h. The first dose of each drug was administered 15 min before commencing the chemotherapy. Nausea, vomiting, anorexia and any additional unpleasant symptoms were recorded on diary cards for the 5 days following therapy. Response was graded in the following manner: complete response (CR), no emetic episodes; major response (MR), 1-2 emetic episodes; minor response (mR), 3-5 emetic episodes; fail (F), more than five emetic episodes. An emetic episode was defined as either a vomit (any episode productive of liquid) or as 1-5 retches within a 5 min period where a retch is a 'vomit' not productive of liquid. Statistical analysis was by comparison of proportions using paired data (Armitage & Berry, 1987).

Fourteen patients were entered in the study between June and October 1988 and their characteristics are shown in Table II. Ten received cisplatin based chemotherapy at a dose of 75 mg $m^{-2}$. Nine patients (64%) had a complete response with no nausea or vomiting. A further one patient had a major response and three a minor response. One patient failed. Using each patient as their own control the CR + MR rate of 71% for the combination was highly significant ($P = 0.001$). In addition to control of nausea and vomiting it was striking that the patients achieving a complete response felt entirely well with no loss of appetite during their chemotherapy. The only side-effect noted was mild headache in two patients. No steroid related toxicity was seen.

**Table I** Chemotherapy schedules

| | No of Patients | Results | | | |
|---|---|---|---|---|---|
| | | *CR* | *MR* | *mR* | *F* |
| Etoposide + cisplatin | 7 | 5 | 1 | 0 | 1 |
| Etoposide + ifosfamide + cisplatin | 1 | | | 1 | |
| Etoposide + vincristine + cisplatin | 1 | | | 1 | |
| Cisplatin | 1 | 1 | | | |
| Etoposide + methotrexate + actinomycin-D | 2 | 2 | | | |
| Adriamycin + cyclophosphamide | 1 | 1 | | | |
| Adriamycin + bleomycin + vincrintine + DTIC | 1 | | | 1 | |

CR, complete response; MR, 1-2 emetic episodes; mR, 3-5 emetic episodes; F, >5 emetic episodes.

**Table II** Patient characteristics

| | |
|---|---|
| Total | 14 |
| Age, median (range) | 28 (18-41) |
| Tumour type: | |
| Choriocarcinoma | 8 |
| Teratoma | 3 |
| Lymphoma | 2 |
| Medulloblastoma | 1 |
| Refractory to Ondansetron | 14 |
| Refractory to dexamethasone | 14 |

Early clinical trials have confirmed the effectiveness of 5HT3 antagonists in controlling the emesis associated with cytotoxic chemotherapy (Cunningham *et al.*, 1987; Kriss *et al.*, 1988; Smith *et al.*, 1990). However, there remain some patients who are inadequately controlled by these agents used alone despite the maintenance of pharmacological serum levels of drug (Smith *et al.*, 1990). It is likely that in such patients several different mechanisms are responsible for inducing vomiting and therefore in order to achieve optimum symptomatic control combinations of anti-emetics with separate modes of action are required. For this study dexamethasone was chosen for use in conjunction with Ondansetron because it is known to be an effective anti-emetic (Kris *et al.*, 1985) and although the precise mechanism of action of dexamethasone is unknown there is no evidence that it interacts with dopaminergic or 5HT3 receptors. In addition, when used for short periods it is largely free from side-effects.

This study shows that 90% of patients failing both dexamethasone and single agent Ondansetron responded to the combination of the two drugs suggesting major synergism between these agents. Cunningham *et al.* (1989) have also made this observation.

Ondansetron plus dexamethasone is an easily administered, non-toxic regimen which is effective in controlling emesis in patients refractory to conventional therapy. A randomised trial is now in progress to confirm whether this combination is active in patients treated with cisplatin at doses of 100-120 mg $m^{-2}$.

Correspondence: D.B. Smith.
Received 19 July 1989; and in revised form 20 October 1989.

### References

ARMITAGE, P. & BERRY, G. (1987). *Statistical Methods in Medical Research*. Blackwell Scientific Publications: Oxford.

COSTALL, B., DOMENEY, A.M., NAYLOR, R.J. & TATTERSALL, F.O. (1986). 5-Hydroxytryptamine M-receptor antagonism to prevent cisplatin induced emesis. *Neuropharmacology*, **25**, 959.

CUNNINGHAM, D., HAWTHORN, J., POPLE, A., GAZET, J.-C., FORD, H.J. & CHALLONER, T. (1987). Prevention of emesis in patients receiving cytotoxic drugs by GR38032F, a selective 5HT3 antagonist. *Lancet*, **i**, 1461.

CUNNINGHAM, D., TURNER, A., HAWTHORN, J. & ROSIN, D. (1989). Ondansetron with and without dexamethasone to treat chemotherapy induced emesis. *Lancet*, **i**, 1323.

KRIS, M.G., GRALLA, R.J., CLARK, R.A. & TYSON, L.B. (1988). Dose Ranging evaluation of the serotonin antagonist CR-C507/75 (GR38032F) when used as an anti-emetic in patients receiving anti-cancer chemotherapy. *J. Clin. Oncol.*, **6**, 659.

KRIS, M.G., GRALLA, R.J., TYSON, L.B. & 6 others (1985). Improved control of cisplatin induced emesis with high-dose metoclopramide and with combinations of metoclopramide, dexamethasone and diphenhydramine. Results of consecutive trials in 255 patients. *Cancer*, **55**, 527.

MINER, W.D. & SANGER, G.L. (1986). Inhibition of cisplatin-induced vomiting by selective 5-hydroxytryptamine M-receptor antagonism. *Br. J. Pharmacol.*, **88**, 497.

SMITH, D.B., NEWLANDS, E.S., RUSTIN, G.J.S., BEGENT, R.H.J., CRAWFORD, S.M. & CARRUTHERS, L.A. (1990). A phase I/II study of GR38032F as anti-emetic prophylaxis against cisplatin-induced nausea and vomiting. *Cancer Chemother. Pharmacol.* (in the press).